# The Novel Peptide Chm-273s Has Therapeutic Potential for Metabolic Disorders: Evidence from In Vitro Studies and High-Sucrose Diet and High-Fat Diet Rodent Models

**DOI:** 10.3390/pharmaceutics14102088

**Published:** 2022-09-30

**Authors:** Nikita A. Mitkin, Vsevolod V. Pavshintcev, Iuliia A. Sukhanova, Igor I. Doronin, Gennady A. Babkin, Marianna Sadagurski, Anton V. Malyshev

**Affiliations:** 1 Metabico Inc., Newton, MA 02460, USA; 2 Department of Biological Sciences, Integrative Biosciences Center (IBio), Wayne State University, Detroit, MI 48202, USA

**Keywords:** novel treatment, peptide drugs, insulin signaling, central action, prediabetes

## Abstract

The aim of this study was to develop a novel peptide potentially applicable for the treatment of metabolic conditions, such as obesity and type 2 diabetes (T2D). We identified CHM-273S from the list of peptides from milk hydrolysate obtained by HPLC/MS-MS. In vitro analysis of primary murine fibroblasts indicated the potential of CHM-273S to upregulate IRS2 mRNA expression. CHM-273S showed a prominent anorexigenic effect in mice with the induction of a key mechanism of leptin signaling via STAT3 in the hypothalamus as a possible effector. In the animal model of metabolic disease, CHM-273S alleviated glucose intolerance and insulin resistance, and induced phosphorylation of Akt at Ser473 and Thr308 in the hepatocytes of high-sucrose diet-fed rats. In a murine model of T2D, CHM-273S mitigated high-fat diet-induced hyperglycemia and insulin resistance and improved low-grade inflammation by diminishing serum TNFα. Mice treated with chronic CHM-273S had a significant reduction in body weight, with a lower visceral fat pad weight and narrow adipocytes. The effects of the peptide administration were comparable to those of metformin. We show the potential of CHM-273S to alleviate diet-induced metabolic alterations in rodents, substantiating its further development as a therapeutic for obesity, T2D, and other metabolic conditions.

## 1. Introduction

Peptides represent a unique class of compounds positioned between small molecules and proteins, yet are biochemically and therapeutically distinct from both. Bioactive peptides possess hormone- or drug-like activity through binding to specific receptors on target cells, leading to the induction of physiological responses [1]. Numerous studies have addressed the versatility of physiological effects produced by peptides, including but not limited to control of high blood pressure, immunomodulation, proliferation regulation, cholesterol reduction, and control of appetite and metabolism [1,2,3,4,5]. The structural versatility of peptides together with their superior safety and tolerability profiles make them promising drug candidates [6].

In multiple studies, it is reported that *Bos taurus* milk protein hydrolysates provide beneficial metabolic effects, including improved glycemic control [7,8,9], enhanced glucose utilization [10,11], and optimized lipid metabolism [12]. Individual whey-derived peptides are reported to induce glucose uptake [13], ameliorate insulin resistance [14], target key diabetes/obesity-associated enzymes [15], and stimulate satiety signaling pathways [16]. These data indicate milk protein hydrolysate as a promising source of novel bioactive peptides applicable for correction of metabolic disorders.

Obesity, metabolic syndrome, and type 2 diabetes mellitus (T2D) are three interrelated conditions that share a number of pathophysiological mechanisms and that are frequently observed to lead, in succession, to cardiovascular diseases (CVD) [17]. There are several Food and Drug Administration (FDA)-approved treatments for T2D, including the first-line treatment metformin (Glucophage) [18] and the novel drug tirzepatide (Mounjaro) [19], a glucagon-like peptide-1 (GLP-1) and glucose-dependent insulinotropic polypeptide (GIP) receptor agonist [20,21]. Effective anti-obesity drug therapies restrict energy intake or increase energy expenditure by acting either centrally or peripherally [22]. The FDA has approved five drugs for long-term use in obesity and overweight: orlistat (Xenical, Alli), phentermine-topiramate (Qsymia), naltrexone-bupropion (Contrave), liraglutide (Saxenda), and semaglutide (Wegovy). Of particular interest is the recently approved sixth medication, setmelanotide (Imcivree), an eight-amino-acid cyclic peptide analog of the endogenous MC4 receptor ligand alpha melanocyte-stimulating hormone (α-MSH). Its use is limited to people who have been diagnosed with one of three specific rare genetic disorders caused by proopiomelanocortin (POMC), proprotein convertase subtilisin/kexin type 1 (PCSK1), or leptin receptor (LEPR) deficiency. Fat cell-derived leptin stimulates the expression of α-MSH via POMC, which regulates hunger, satiety, and energy expenditure by binding to MC4 receptors in the hypothalamus [23]. POMC expression is upregulated by leptin throughout the Janus kinase 2 (JAK2)–signal transducer and activator of transcription factor 3 (STAT3) pathway, where the key event is phosphorylation and nuclear translocation of STAT3 [24]. This mechanism of POMC production is involved in the central action of other substances controlling Gαs–cyclic adenosine monophosphate (cAMP) signaling, such as GLP-1 receptor agonists [25] and amylin receptor 2 (AMY2) agonists [26].

Because drugs for T2D and obesity are administered chronically, they must be risk-free. However, the current treatment options have safety drawbacks. The identification of compounds with adequate safety and tolerability profiles and the same or greater efficacy than that of standard treatments would have strong clinical potential. The close association between obesity and insulin resistance, and their progression to T2D, is a serious health problem. The regulation of distinct downstream components of the insulin signal transduction pathway might be a good starting point for the management of various metabolic conditions. The activated insulin receptor phosphorylates insulin receptor substrate (IRS) proteins on multiple tyrosine residues serve as docking sites for downstream mediators of metabolic actions, including phosphatidylinositol-3 kinase (PI3K) [27]. A promising treatment approach for T2D is the regulation of insulin signaling by either amelioration of IRS2 synthesis [28] or activation of its downstream PI3K/AKT pathway [29]. AKT phosphorylates many substrates, including proteins regulating cell survival, growth and differentiation (in particular, adipocytes), gene expression, and glycogen synthesis [28]. Animal studies have shown an increased appetite, lean and fat body mass, linear growth, and insulin resistance that progressed to diabetes when Irs2 was conditionally knocked out in the pancreas β cells and the hypothalamus of mice [30]. Amelioration of IRS2 expression and subsequent induction of PI3K/AKT signaling underlie improved insulin secretion, increased insulin sensitivity, and better appetite regulation reported in both diabetic humans and rodents treated with GLP-1 or exendin-4 [30,31]. Because Irs2 function is inhibited by serine phosphorylation and ubiquitin-mediated degradation that is mediated by proinflammatory cytokines [32], effective management of chronic inflammation might prevent the progression of insulin resistance to diabetes [30].

Insulin resistance and impaired insulin secretion, which are characteristics of T2D, can be modeled in animals by using different diets, such as high-sucrose, high-fat, or cafeteria (high-fat and high-sucrose) diets, or by direct genetic disruption [33]. There is considerable epidemiological evidence suggesting that intake of added sugars and/or sugar-sweetened beverages is associated with the development and/or prevalence of fatty liver, dyslipidemia, insulin resistance, hyperuricemia, CVD, and T2D, and many of these associations are independent of body weight gain or total energy intake [34]. High-sucrose diet (HSD) is a time-efficient model widely used in rats for induction of whole body insulin resistance, associated with elevated serum glucose and insulin levels [35]. Diets rich in fat not only induce obesity in humans [36,37,38,39], but can also make animals obese [40,41]. The C57Bl/6 mouse is a particularly effective model to mimic human metabolic derangements that are observed in obesity [42]. They develop a full manifestation of obesity after 16 weeks of a high-fat diet (HFD), including adipocyte hyperplasia, mesenteric fat deposition, increased fat mass, diabetes, and hypertension [43].

In the current study, we describe the discovery of a novel peptide CHM-273S and evaluate its therapeutic potential in HSD and HFD animal models of metabolic alterations.

## 2. Materials and Methods

### 2.1. Peptide Drug Candidates

We used high-performance liquid chromatography–tandem mass spectrometry (HPLC/MS-MS) to separate proprietary *Bos taurus* milk hydrolysates into protein fractions. The major peptides of these fractions were identified and annotated manually. We selected 14 peptides (Table 1) derived from milk proteins αs(1/2)-casein (CasA1/2), β-casein (CasB), κ-casein, β-lactoglobulin (LacB), glycosylation-dependent cell adhesion molecule 1 (GlyCam1), osteopontin (OSTP), and polymeric immunoglobulin receptor (PIGR) for their novelty and lack of any previous reports on their functions. We tested the selected peptides as well as CHM-273 pharmacophores (Table 1) for in vitro activity in primary murine fibroblasts. Peptides (98% purity) were commercially synthesized by Syneuro LLC (Moscow, Russia) for in vitro studies and by Peptide 2.0 Inc. (Herndon, VA, USA) for in vivo studies.

### 2.2. Animals, Treatments, and Tissue Processing

In total, 190 male C57BL/6 mice and 50 male Sprague Dawley rats were used in the study. The animals were housed under constant environmental conditions (12-h photoperiod at 22 ± 2 °C) with ad libitum access to food and water. Animal experiments were conducted in accordance with the European Directive 2010/63/EU of the European Parliament (Council of 22 September 2010 on the protection of animals used for scientific purposes), the European Directive 2004/10/EC of the European Parliament (Council of 11 February 2004 on the protection of animals used for scientific purposes), and the Russian “GOST 33216-2014” Guidelines for the maintenance and care of laboratory animals. All animal experiments were approved by the local Bioethics Commissions of Institute of Mitoengineering of MSU LLC, Pushchino Branch of IBCh RAS, and the Koltzov Institute of Developmental Biology RAS. All manipulations with animals were carried out at the end of the 14-day adaptation period.

This paper describes five experiments: the first is an evaluation of the in vitro effects of novel peptides, the second is an evaluation of feeding behavior in naïve mice, the third is an evaluation of the peptide ability to induce STAT3 signaling in the hypothalamus of naïve mice, the fourth involves an evaluation of an HSD in rats, and the fifth involves an evaluation of an HFD in mice.

In the first series of experiments, fibroblasts were isolated from skin tissues of mice (*n* = 20) according to the standard protocol [44]. Peptides (Table 1) were diluted in saline and added to the incubation medium to the final concentration of 0.005, 0.05, or 0.5 mg/mL. CHM-273 pharmacophores were added at 100 μM, which corresponds to an efficient CHM-273 dose of 0.05 mg/mL. Each treatment group was represented with two biological replicates.

In the second series of experiments, mice (*n* = 30) were administered 5 mg/kg CHM-273S intranasally (i.n.), 1 mg/kg leptin intraperitoneally (i.p.) (Sigma-Aldrich Inc., Saint Louis, MO, USA), or with vehicle alone (i.p.) right before testing. Both the design of the experiment and the dose of leptin were chosen according to the recent study [45]. The experiment was performed by the Institute of Mitoengineering of MSU LLC (Moscow, Russia) on a contract basis. The Institute of Mitoengineering of MSU LLC is accredited by Rus-LASA, a member of the Federation of European Laboratory Animal Science Associations (FELASA), which indicates that all the studies were performed in conformity with standards defined by the Directive 2010/63/EU of the European Parliament and of the Council of 22 September 2010 on the protection of animals used for scientific purposes.

In the third series of experiments, for immunohistochemistry (IHC) assays, mice (*n* = 15) were treated with 10 mg/kg CHM-273S (i.n.), 5 mg/kg leptin (i.p.) [46], or vehicle (i.p.). Mice were euthanized 60 min after drug administration and 24-hour food deprivation with the combination of 20 mg/kg Zoletil (Virbac, Carros, France) and 5 mg/kg Rometar (Bioveta Inc., Ivanovice na Hané, Czech Republic). The fixation procedure included transcranial perfusion with saline, followed by 10% formalin. After the animals had been decapitated, their brains were dissected and fixed in 10% formalin, then washed in phosphate-buffered saline (PBS) for 3 × 30 min and placed in 30% sucrose in PBS overnight until the tissue was completely impregnated. After fixation, hypothalamuses were separated from the rest of the brain with a scalpel, the samples were placed in a mold, and freezing medium was poured over the samples. Then, the samples were frozen in liquid nitrogen vapor and 10 µm slices were cut using Microm HM525 cryostat (Thermo Fisher Scientific, Waltham, MA, USA). The slices were immediately mounted on slides. Overall, 4–5 glasses with 8 slices on each were obtained from each brain. The experiment was performed by the Koltzov Institute of Developmental Biology RAS (Moscow, Russia) on contract basis in accordance with the Russian “GOST 33216-2014” Guidelines for the maintenance and care of laboratory animals.

In the fourth series of experiments, rats (*n* = 45) were provided with an additional drinker containing 30% sucrose in water for 5 weeks (HSD), or an additional drinker with water (*n* = 5, “control” group). The animals’ weights were measured once every 2 days from day 8 of HSD administration. The glucose concentration was measured once a week after a 12-hour fast using an ACCU-CHEK Active glucometer (Roche, Indianapolis, IN, USA) in the blood collected from the tail vein. Sucrose consumption was evaluated by weighing the drinker every other day, taking the average per cage. The development of metabolic syndrome in rats was considered when the blood glucose concentration had increased significantly for two consecutive measurements relative to the values in the control group. It is important to note that this model mimics metabolic syndrome due to impaired carbohydrate metabolism with insulin resistance, which has been reported in other studies [47,48,49]. For the oral glucose tolerance test (OGTT), CHM-273S was administered i.p. at 1 or 10 mg/kg 0, 2, 12, or 24 h before the test. Control animals received i.p. saline. The animals were kept on an HSD for another week. Twenty-four rats were sacrificed for serum insulin and plasma glucose determination and 26 for Western blot (WB) analysis of liver samples. The blood and liver samples were collected 2 or 12 h after CHM-237S treatment (1 or 10 mg/kg, i.p.). Trunk blood was collected after decapitation, centrifuged, and the serum was aliquoted and stored at −20 °C until analysis. To obtain liver samples, rats were subjected to anesthesia with a combination of 25 mg/kg Zoletil and 15 mg/kg xylazine. The animals were injected with insulin (10 U/kg, 100 μL/kg body weight) (Kanisulin, Intervet International GmbH, Unterschleißheim, Germany) into the inferior vena cava. Three minutes later, liver samples (around 100 mg) were dissected, snap-frozen in liquid nitrogen, and stored at −80 °C. Then, the animals were euthanized by an overdose of Zoletil/xylazine. This part of the study was performed by the Institute of Mitoengineering of MSU LLC (Moscow, Russia) on a contract basis.

In the fifth series of experiments, mice were kept on either the HFD (SSNIFF R/M-H V1534-30 with the addition of lard (35% carbohydrates, 45% fat, and 20% protein; 516 kcal/100 g), *n* = 100) or regular chow (“control” group, *n* = 25) for 16 weeks. The animals’ weights were monitored weekly. For the OGTT, CHM-273S was administered i.p. or i.n. at 5 mg/kg. The drug of comparison was metformin (JSC Rafarma, Moscow, Russia) administered perorally (p.o.) at 250 mg/kg, 2 or 12 h prior to the test. Control animals received i.p. saline, administered similarly to all drug-treated groups to control for stress in the injection procedures. After the OGTT, groups of 10 animals with approximately the same body weight were formed; they received 5 mg/kg CHM-273S i.p. or i.n., or 250 mg/kg metformin p.o. for 4 weeks along with the HFD. The animals’ weights were monitored every other day. The day after the last treatment, the animals were euthanized with Zoletil/xylazine for necropsy. Terminal blood samples were collected from the inferior vena cava, centrifuged, and serum was aliquoted and stored at −20 °C until analysis. The internal organs were examined for the presence of macro-damage and weighed. The adipose tissue surrounding the epididymis was evaluated as a marker of obesity in animals [50]. For histological examination, samples of adipose tissue were fixed in 10% formalin. This series of experiments was performed by the Laboratory of Biological Testing, Pushchino Branch of IBCh RAS (Pushchino, Russia) on a contract basis. The laboratory is accredited by the Association for Assessment and Accreditation of Laboratory Animal Care International (AAALAC International) and is officially recognized as conforming to the principles of good laboratory practice (GLP) by the Slovak National Accreditation Service (Statement of GLP Compliance # G-044), which indicates conformity with GLP according to the Act No. 67/2010 Coll., the OECD Principles of GLP, and Directive 2004/10/EC of European Parliament and of the Council.

### 2.3. Feed

All animals initially received a standard diet (SSNIFF R/M-H V1534-30 (58% carbohydrates, 9% fat, and 33% protein; 306 kcal/100 g)). For the HFD groups, the high-fat feed was used. One kilogram of feed contained 610 g of ground SSNIFF feed and 360 g of melted pork lard, water (≈250 mL, heated to 60–70 °C), 10 g of sodium chloride, and 30 g of sodium glutamate. The mixture was brewed to a dough consistency, and food granules were formed. Then, the granules were dried at 60–70 °C for 10–12 h. The finished product was transferred to the animal-keeping area. The prepared food was stored at 4 °C for no more than 7 days. The approximate energy value of the HFD feed was 516 kcal/100 g (35% carbohydrates, 45% fat, and 20% protein).

### 2.4. Cell Culture

Fibroblasts were cultured in complete DMEM/F12 medium containing 15% fetal bovine serum (FBS) for 2 days. For gene expression analysis, cells were incubated with individual peptides (from Table 1) in the same medium for 24 h.

### 2.5. RNA Isolation and Reverse Transcription–Quantitative Polymerase Chain Reaction (RT-qPCR) Protocol

After 24 h incubation with peptides, the cells were treated with the ExtractRNA reagent (Evrogen, Moscow, Russia), an analog of Trizol, and RNA was isolated according to the manufacturer’s protocol. The RNA concentration and 260/280 ratio were measured with a DS-11 spectrophotometer (DeNovix, Wilmington, DE, USA). To obtain complementary DNA (cDNA), total RNA was reverse transcribed using the MMLV-RT Kit with the oligo-dT primer (Evrogen, Moscow, Russia).

Messenger RNA (mRNA) was quantified using a 7500 real-time PCR System (Applied Biosystems, Waltham, MA, USA). The reactions included qPCR mix-HS-SYBR + Low Rox assay (Evrogen, Moscow, Russia) and were run in duplicates. All primer sequences were generated and verified for specificity by Primer-BLAST (NCBI-NIH). The sequences of the primers used were as follows: *Tsc1* (Fw—TTATCCATCCTCTCGCTGCT, Rv—AGGTGCTGCTTCCCTGACT); *Tsc2* (Fw—ATGGATGTTGGCTTGTCCTC, Rv—TAAGCAGTTGTAGCAGACCA); *Rps6kb1* (Fw—GACATGGCAGGAGTGTTTGA, Rv—TTTCCATAGCCCCCTTTACC); *Akt* (Fw—TCTATGGTGCGGAGATTGTG, Rv—CTTGATGTGCCCGTCCTTGT); *Irs2* (Fw—CGGCCTCAACTATATCGCCA, Rv—GCGCTTCACTCTTTCACGAC); *Stat3* (Fw—CTTGTCTACCTCTACCCCGACAT, Rv—GATCCATGTCAAACGTGAGCG); *Srebp1* (Fw—ATCGCAAACAAGCTGACCTG, Rv—AGATCCAGGTTTGAGGTGGG); *RPL27* (Fw—AAGCCGTCATCGTGAAGAACA, Rv—CTTGATCTTGGATCGCTTGGC). The relative gene expression was determined as the ratio of the target gene to the internal reference gene expression (*Rlp27*) based on the threshold cycle (Ct) values using QGENE software.

### 2.6. Immunohistochemistry of Phosphorylated STAT3 (p-STAT3) in Mouse Hypothalamus

The hypothalamus sections were fixed in 10% formalin (10 min), washed in PBS (5 × 8 min), and then incubated with 1% H_2_O_2_ and 0.3% NaOH (20 min), 0.3% glycine (10 min), and 0.03% sodium dodecyl sulfate (SDS; 10 min). After rinsing in PBS, the sections were blocked with 10% normal goat serum (in 0.3% Triton X-100; 60 min) and incubated overnight at 4 °C with the primary p-STAT3 (Y705) antibody (Cell Signaling Technology, Boston, MA, USA), diluted 1:300 in a block solution. After thorough rinsing in PBS (3 × 8 min), the sections were incubated with secondary antibody conjugated to Alexa Fluor 488 (Thermo Fisher Scientific, Waltham, MA, USA) diluted 1:600 in block solution overnight at 4 °C. Then, the preparations were washed in PBS (3 × 8 min) and mounted in Vectashield mounting medium with DAPI (Vector Labs, Burlingame, CA, USA) under a cover glass. The sections were viewed using an inverted microscope Eclipse Ni (Nikon, Tokyo, Japan), and pictures were taken. The percentage of p-STAT3-positive nuclei to the total number of nuclei was calculated using ImageJ software (NIH).

### 2.7. Serum Glucose, Insulin, and Homeostatic Model Assessment for Insulin Resistance (HOMA-IR)

The insulin concentration in the serum samples of animals was measured using enzyme-linked immunosorbent assay (ELISA) kits (Rat Insulin ELISA [10-1250-01] or Mouse Insulin ELISA [10-1247-01], Mercodia, Uppsala, Sweden), according to the manufacturer’s protocols. Glucose was measured with an automated biochemical analyzer (BioSystems, Barcelona, Spain) using a standard analytical kit, according to the manufacturer’s protocol. The estimate of insulin resistance by HOMA-IR was calculated with the formula:

$$\left(Fasting\;glucose\;\left[mM\right]\times Fasting\;insulin\left[mUL^{-1}\right]\right)\;/\;22.5$$

### 2.8. WB Analysis

For WB analysis, tissues were lysed and resolved with 10% sodium dodecyl sulfate–polyacrylamide gel electrophoresis and transferred onto a polyvinylidene difluoride (PVDF) membrane (Millipore, Billerica, MA, USA). The following primary antibodies were used: p-Akt (Ser473) (D9E), p-Akt (Thr308) (D25E6), phosphorylated ribosomal protein S6 kinase beta-1 (p-p70S6K; Thr421/Ser424), and GAPDH (14C10) (1:1000) (all Cell Signaling Technology, Boston, MA, USA). A horseradish peroxidase-conjugated anti-rabbit IgG secondary antibody (1:5000) was used (Cell Signaling Technology, Boston, MA, USA). The results were normalized to the reference protein GAPDH. The analysis was carried out using Image Lab 5.2.1 software (Bio-Rad Inc., Hercules, CA, USA).

### 2.9. Evaluation of the Proinflammatory Cytokine Tumor Necrosis Factor Alpha (TNFα) in Serum

The concentration of TNFα was measured in serum samples using a mouse ELISA kit (Invitrogen, Waltham, MA, USA), according to the manufacturer’s protocol. Analysis was performed using a Multiskan GO spectrophotometer (Thermo Fischer Scientific, Waltham, MA, USA).

### 2.10. Free-Feeding Food Intake Experiment in Mice

Before the experiment, mice were pre-weighed and separated into three groups based on their weight. The study was conducted over 2 days. On day 1, the animals were adapted to the experimental conditions. For this, each mouse was placed in a standard T3 home cage with an empty Petri dish inside for 90 min. The animals were deprived of food overnight. On day 2, a Petri dish was filled with two pre-weighed food pellets, and after drug injection, mice were placed in a cage for 90 min. After 30, 60, and 90 min, the food from the cage was weighed. The home cage conditions as well as preliminary adaptation allowed us to evaluate the food motivation of the animals under standard conditions, without affecting the anxiety component of a novel environment [51].

### 2.11. OGTT

After a 12 h overnight fast and baseline sampling, a 2 g/kg glucose solution (40%) was administered orally by gavage, followed by blood collection after 30, 60, 90, and 120 min from the tail for blood glucose determination using ACCU-CHEK Active glucometer (Roche, Indianapolis, IN, USA). The glucose levels at baseline and 120 min after the glucose load was considered to calculate the area under the curve (AUC) of the OGTT.

### 2.12. Histology

Histological analysis of liver and adipose tissue was performed for all euthanized animals. For this, the fixed tissues were dehydrated and then embedded in paraffin; the paraffin blocks were then cut into sections. The sections were stained with hematoxylin and eosin following a standard protocol. Tissue specimens were examined by light microscopy. Microscopic analysis was performed using a DMLA Leica transmitted light microscope (Leica Microsystems GmbH, Wetzlar, Germany), a Photometrics Cool SNAP cf video camera (Teledyne Photometrics, Tucson, AZ, USA), and Mekos software (Moscow, Russia). The estimated parameters were the linear size of visceral fat adipocytes (µm) and the subcutaneous fat thickness (µm). For each animal, 100 adipocytes were measured on sections of the visceral fat samples in 15–20 random fields of view.

### 2.13. Statistical Analysis

Statistical analysis was performed using Prism 9.1 (GraphPad, San Diego, CA, USA). The normality of the data was assessed using the Shapiro–Wilk test. Normally distributed data were analyzed by one-way/repeated measures analysis of variance (ANOVA) with the Holm–Šídák post hoc test. The data are presented as the mean ± standard error of the mean (SEM). In vitro screening experiments were carried out in two biological and two technical replicates, and analyzed by nested one-way ANOVA, followed by the two-stage linear step-up procedure of Benjamini, Krieger, and Yekutieli. The data are presented as individual values and the median. Non-normally distributed data were analyzed by the Kruskal–Wallis test, followed by Dunn’s post hoc test. The data are presented as box and whisker plots. The AUC of the OGTT was calculated by trapezoidal approximation of plasma glucose levels with GraphPad Prism. Differences between groups were considered to be significant at *p* < 0.05.

## 3. Results

### 3.1. In Vitro Screening for Novel Regulatory Peptides to Treat Metabolic Disorders

**Figure 1:**
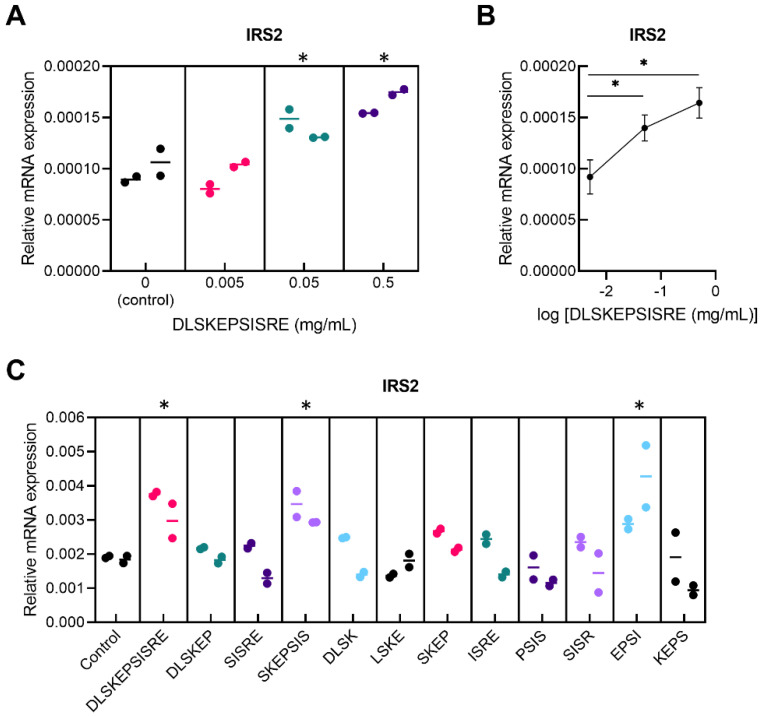
Enhanced *Irs2* messenger RNA (mRNA) transcription in primary murine fibroblasts after incubation with DLSKEPSISRE and its pharmacophores SKEPSIS and EPSI. (**A**,**B**) DLSKEPSISRE dose-dependently increased *Irs2* mRNA levels in cell culture, with a significant effect at 0.05 and 0.5 mg/mL (F_(3, 4)_ = 11.98, *p* = 0.018). (**C**) SKEPSIS and EPSI pharmacophores (0.05 mg/mL) increased *Irs2* mRNA levels in a similar manner to DLSKEPSISRE (F_(12, 13)_ = 3.276, *p* = 0.022). The results are presented as individual values (dots) and the median (dash) of two biological and two technical replicates. * *p* < 0.05 versus the control group; nested one-way analysis of variance, discovery by two-stage linear step-up procedure of Benjamini, Krieger, and Yekutieli, at a Q threshold of 0.05.

We explored the suitability of milk hydrolysate-derived peptides as a source of novel active compounds for the treatment of metabolic disorders. Our expectations for the presence of functional peptides in the original substance were based on the fact that the treatment of cultured murine fibroblasts with total hydrolysate resulted in beneficial changes in expression levels of the range of genes involved in intracellular insulin receptor signaling (*Irs2* and *Akt*) and other metabolic regulators (*Tsc1, Tsc2, Rps6kb1*, *Stat3*, and *Srebp1*) (Supplementary Figure S1). Although skin is not a classical insulin target tissue, the rationale of the use of skin fibroblasts for the screening corresponds to the essential role of insulin receptor signaling in normal skin cell physiology [52] and to the presence of insulin signaling pathways typical for classical insulin-sensitive cells in skin fibroblasts [53,54]. To begin, we obtained a list of peptides from bovine milk hydrolysate using HPLC-MS/MS. For the screening, we manually selected 14 novel peptides that originated from milk proteins—CasA1/2, CasB, CasK, LacB, GlyCam1, OSTP, and PIGR—and tested if they could potently regulate metabolism-related intracellular processes in primary murine fibroblasts. The preliminary study revealed that none of the peptides affected the transcription of components of the mammalian target of rapamycin (mTOR) signaling pathways (*Tsc1*, *Tsc2*, and *Rps6kb1*) or regulators of carbohydrate and lipid metabolism (*Akt*, *Stat3*, and *Srebp1*). Among the tested peptides, only DLSKEPSISRE, a fragment of the GlyCam1 protein, potently enhanced transcription of *Irs2*, a key regulator of the insulin signaling pathway. A dose-finding study revealed that DLSKEPSISRE dose-dependently enhanced *Irs2* mRNA expression, with a significant effect at 0.05 and 0.5 mg/mL (Figure 1A,B). We then divided DLSKEPSISRE into tetrapeptide fragments using a sliding window with a step of one amino acid, as well as two hexapeptides and one pentapeptide, and tested them for functional activity in primary murine fibroblasts. We found that, similarly to the full-length peptide, SKEPSIS and EPSI peptides enhanced *Irs2* mRNA expression (Figure 1C). We later named DLSKEPSISRE and its pharmacophores SKEPSIS and EPSI as CHM-273, CHM-273L, and CHM-273S, respectively. Because CHM-273 and CHM-273L both include the EPSI fragment, we propose that CHM-273S is responsible for the peptides’ in vitro effects. Thus, for the subsequent experiments with rodent models of metabolic diseases, we used the shortest active fragment, CHM-273S.

### 3.2. Free-Feeding Food Intake and Hypothalamic STAT3 Activity Regulation by CHM-273S

**Figure 2:**
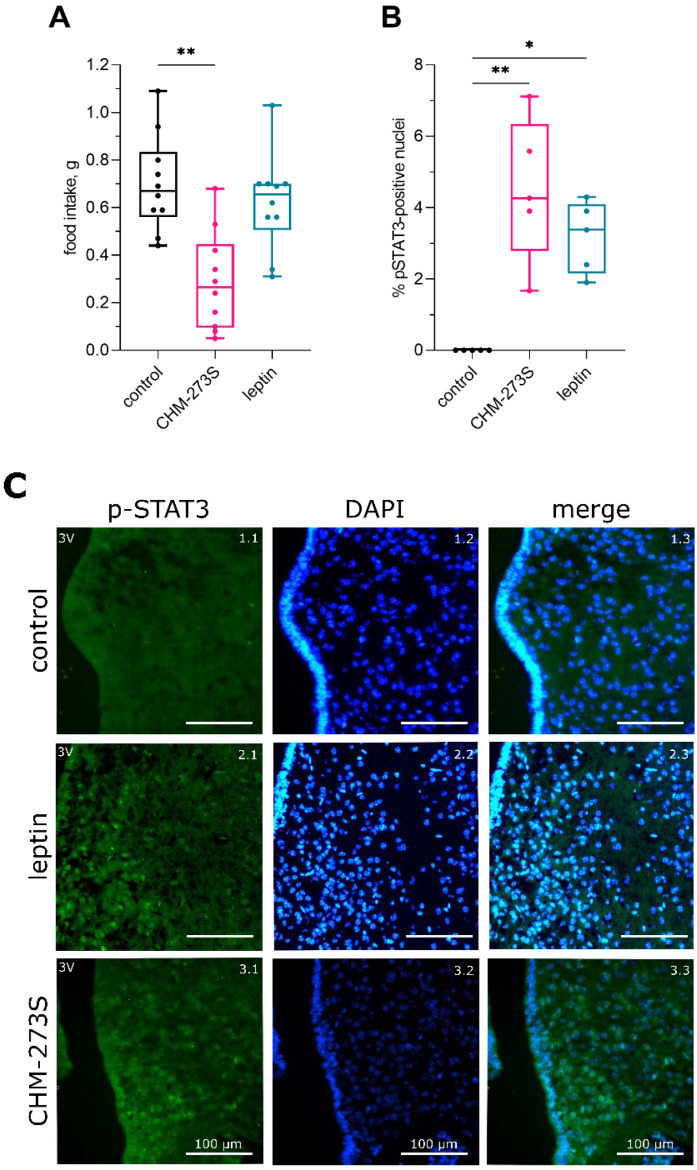
The effects of CHM-273S on food intake and p-STAT3 in the hypothalamus of naïve mice. (**A**) CHM-273S (5 mg/kg intranasally, i.n.) but not leptin (1 mg/kg intraperitoneally, i.p.) significantly reduced 90 min home cage food consumption (H_(3, 30)_ = 13.52, *p* = 0.0012). (**B**) CHM-273S (10 mg/kg i.n.) significantly induced STAT3 phosphorylation in the hypothalamus, similarly to leptin (5 mg/kg i.p.) (H_(3, 15)_ = 10.35, *p* = 0.0007). The results are presented as box and whisker plots with individual values. * *p* < 0.05, ** *p* < 0.01 versus the control group; Kruskal–Wallis test followed by Dunn’s multiple comparison test. (**C**) CHM-273S increased the number of p-STAT3-immunoreactive cells. Representative immunofluorescence photomicrographs of hypothalamic sections are shown under control conditions (1.1–1.3; 60 min after i.p. saline injection), leptin (2.1–2.3; 60 min after i.p. 5 mg/kg leptin), and CHM-273S-stimulated conditions (3.1–3.3; 60 min after 10 mg/kg i.n. CHM-273S). STAT3 immunoreactivity is shown in green (1.1–3.1), nuclear DAPI stain is shown in blue (1.2–3.2), and the overlays of these confocal images are shown in 1.3–3.3.

We tested the appetite of mice in non-stressful home cage conditions. We pre-weighed the mice and separated them into three groups based on weight, with no differences in mean body weight between the control and treatment groups (mean ± SEM = 25.14 ± 0.35 g). CHM-273S administered at 5 mg/kg (i.n.) before the test significantly reduced the amount of food consumed by the animals at the end of the 90-minute experiment (Figure 2A). The rationale of the use of 5 mg/kg as an initial dose for in vivo experiments is based on the previous studies of tetrapeptides missing additional modifications and improved stability profiles [55,56]. This dose is typical for initial studies of the peptides with metabolically relevant features [57,58]. Leptin administered at 1 mg/kg (i.p.) failed to produce a significant effect in this test.

In a separate study, we analyzed p-STAT3 in the hypothalamus of naïve C57BL/6 mice 60 min after i.n. CHM-273S (10 mg/kg), i.p. leptin (5 mg/kg), or i.p. saline (control group) treatment. We found STAT3-positive nuclei in the hypothalamus of mice treated with CHM-273S or leptin, and no expression of p-STAT3 in the control group (Figure 2B,C). We found p-STAT3-immunoreactive nuclei in 4% of cells after treatment with CHM-273S and in 3% of cells after leptin injection (Figure 2B).

### 3.3. Glucose Tolerance, Insulin Resistance, and Liver Insulin Signaling Alterations Induced by the HSD and the Acute CHM-273S Treatment Effects

The body weight of HSD-fed rats did not differ from the control group over the 4-week experiment (Table 2). Blood glucose levels increased after 3 weeks of consuming the HSD and remained significantly elevated over 2 consecutive weeks (days 15–22 and 22–28) in the HSD group (Table 2). We observed the development of hyperglycemia in rats after they had consumed the HSD for 28 days compared with animals fed the standard diet, suggesting reduced insulin sensitivity induced by the HSD in rats.

**Figure 3:**
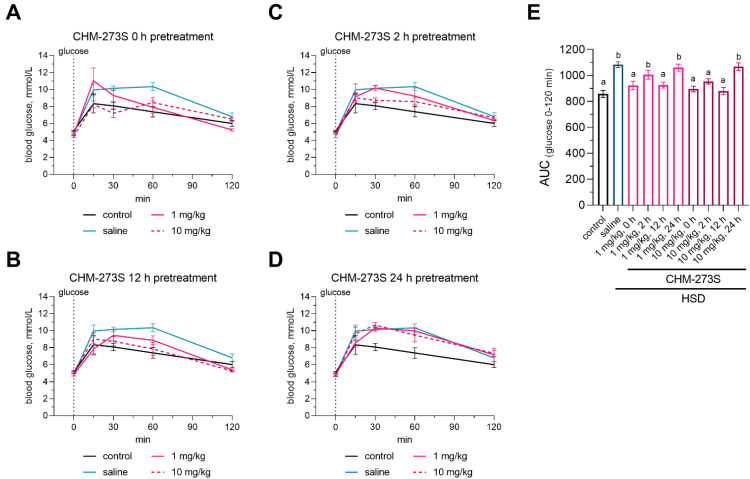
The effects of CHM-273S pretreatment on the oral glucose tolerance test (OGTT) in rats after they had consumed the high-sucrose diet (HSD) for 5 weeks. Blood glucose concentrations were measured by a glucometer after a 12 h fast at 0, 15, 30, 60, and 120 min following gavage with 2 g/kg of an oral glucose (40%) preload (**A**) 0 h, (**B**) 2 h, (**C**) 12 h, or (**D**) 24 h after intraperitoneal (i.p.) CHM-273S (1 or 10 mg/kg) treatment (*n* = 5 each group). (**E**) Area under the curve (AUC) for the OGTT in HSD-fed rats after CHM-273S administration. Blood glucose levels were significantly higher in the HSD-fed, saline-treated group (*n* = 5) and in HSD-fed, CHM-273S-treated rats (1 mg/kg 2 or 24 h or 10 mg/kg 24 h before OGTT, *n* = 5 each) (F_(9,40)_ = 9.46, *p* < 0.0001). There was a significant reduction in blood glucose compared with the HSD-fed, saline-treated rats (*n* = 5) in groups pretreated with 1 mg/kg (0 or 12 h before OGTT, *n* = 5 each) or 10 mg/kg CHM-273S (0, 2, or 12 h before OGTT, *n* = 5 each) (F_(9,40)_ = 9.46, *p* < 0.0001). The results are presented as the mean ± standard error of the mean. Different superscript letters indicate significant differences at *p* < 0.05.; one-way analysis of variance with the Holm–Šídák multiple comparisons test.

We examined the therapeutic potential of a single CHM-273S injection in the OGTT. Glucose intolerance assessed by the OGTT was present after 5 weeks of consuming the HSD (Figure 3A–D for glucose curves for each time point, and Figure 3E for the associated AUC). CHM-273S potently normalized glucose levels when administered at 10 mg/kg i.p. 0, 2, or 12 h before glucose gavage, and when administered at 1 mg/kg, when administered 0 or 12 h before the test (Figure 3A–E).

**Figure 4:**
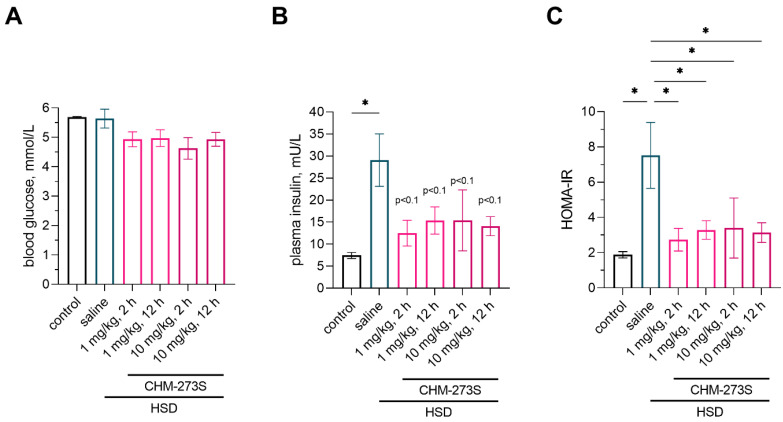
Fasting blood glucose, insulin, and homeostatic model assessment for insulin resistance (HOMA-IR) in high-sucrose diet (HSD)-fed rats pretreated with CHM-273S 2 or 12 h before blood collection. (**A**) Blood glucose levels in HSD-fed, saline-treated (*n* = 4) and HSD-fed, CHM-273S-treated (*n* = 4 each) rats did not differ from the control values (*n* = 4) after a 12 h fast (F_(5,18)_ = 2.17, *p* = 0.10). (**B**) Plasma insulin was significantly higher in HSD-fed, saline-treated rats (*n* = 4) compared with the control group (*n* = 4). Administration of 1 or 10 mg/kg CHM-273 intraperitoneally (i.p.) 2 or 12 h before blood collection (*n* = 4 each) showed a trend (*p* < 0.1) for reduced insulin levels compared with the HSD-fed, saline-treated group (F_(5,18)_ = 2.67, *p* = 0.05). (**C**) The HOMA-IR index was restored to the control values in HSD-fed rats when treated with 1 or 10 mg/kg CHM-273 i.p. 2 or 12 h before blood collection (*n* = 4 each; F_(5,18)_ = 2.94, *p* = 0.04). The results are presented as the mean ± standard error of the mean. * *p* < 0.05 versus the HSD-fed, saline-treated group; one-way analysis of variance with the Holm–Šídák multiple comparisons test.

After the OGTT, the rats were provided 30% sucrose solution for another week. Plasma insulin and HOMA-IR, a marker of insulin resistance, were significantly increased in rats after they had consumed the HSD for 5 weeks (Figure 4B,C); there were no changes in fasting blood glucose levels (Figure 4A). CHM-273 administered at 1 or 10 mg/kg^2^ or 12 h prior to the blood collection tended to reduce blood insulin to the control values and thus significantly normalized HOMA-IR (Figure 4B,C).

**Figure 5:**
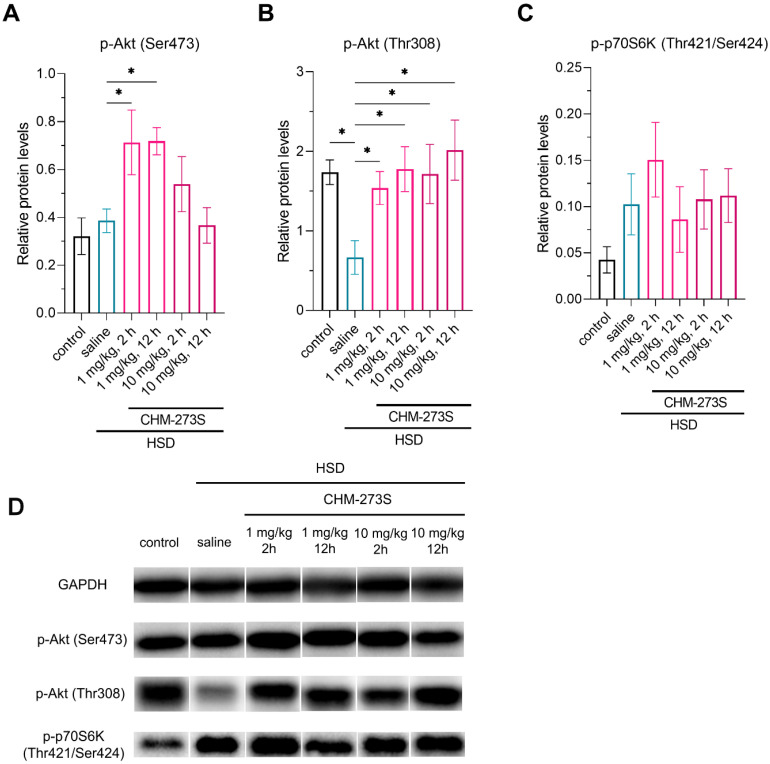
Western blot analysis of p-Akt (Ser473), p-Akt (Thr308), and p-p70S6K (Thr421/Ser424) proteins in the liver of high-sucrose diet (HSD)-fed, CHM-273-treated rats (2 or 12 h prior to sample collection), normalized to GAPDH. Three minutes before the liver sample collection, the animals received intravenous insulin infusion (10 U/kg). (**A**) p-Akt (Ser473) levels were not affected by the HSD, but treatment with CHM-273S (1 mg/kg intraperitoneal, i.p.) 2 or 12 h before sample collection (*n* = 4–5) increased its expression in hepatocytes compared with the HSD-fed, saline-treated group (*n* = 5; F_(5,21)_ = 4.43, *p* = 0.006). (**B**) p-Akt (Thr308) was significantly reduced in HSD-fed, saline-treated rats, while CHM-273S given 2 or 12 h prior to collection at 1 or 10 mg/kg (*n* = 4–5) restored its levels to the control values (*n* = 4; F_(5,21)_ = 2.96, *p* = 0.04). (**C**) p-p70S6K (Thr421/Ser424) levels did not differ among the treatment groups (F_(5,21)_ = 1.09, *p* = 0.32). The results are presented as the mean ± standard error of the mean. * *p* < 0.05 versus the saline-treated group; one-way analysis of variance with the Holm–Šídák multiple comparisons test. (**D**) Representative Western blot bands for each group are shown; GAPDH serves as a loading control.

WB analysis of liver samples revealed that HSD significantly reduced p-Akt (Thr308) but had no effect on p-Akt (Ser473) and p-p70S6K (Thr421/Ser424) in hepatocytes (Figure 5A–D). CHM-273S administration 2 or 12 h prior to sample collection at either dose normalized p-Akt (Thr308) (Figure 5B), and 1 mg/kg CHM-273S increased p-Akt (Ser473) in rat hepatocytes (Figure 5A).

### 3.4. Glucose Tolerance, Insulin Resistance, and Systemic Inflammation in HFD-Fed Mice and the Effects of Chronic CHM-273S Treatment

**Figure 6:**
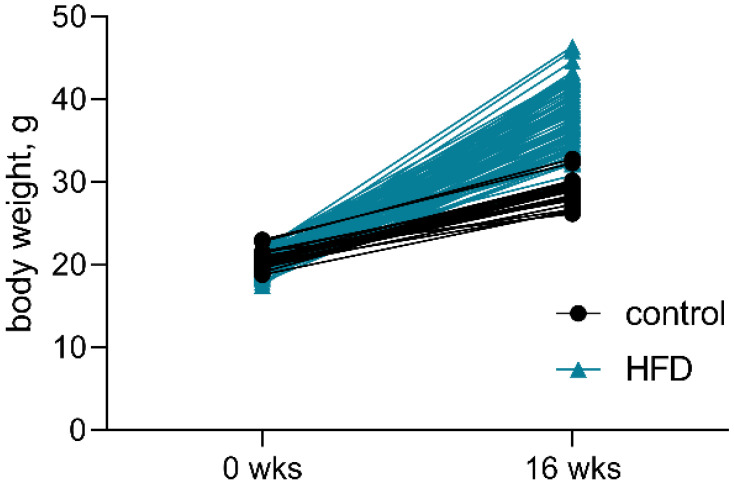
Body weight of mice fed standard chow (control, *n* = 25) or the high-fat diet (HFD, *n* = 100) at the beginning of the experiment (0 weeks (wks)) and after 16 wks. The approximate energy was 516 kcal/100 g (45% fat, 35% carbohydrates, and 20% protein) for the HFD feed and 306 kcal/100 g (9% fat, 58% carbohydrates, and 33% protein) for the standard chow.

Mice fed the HFD for 16 weeks had significantly higher body weight, and the HFD was responsible for 46% excess weight gain in animals (Table 3 and Figure 6).

**Figure 7:**
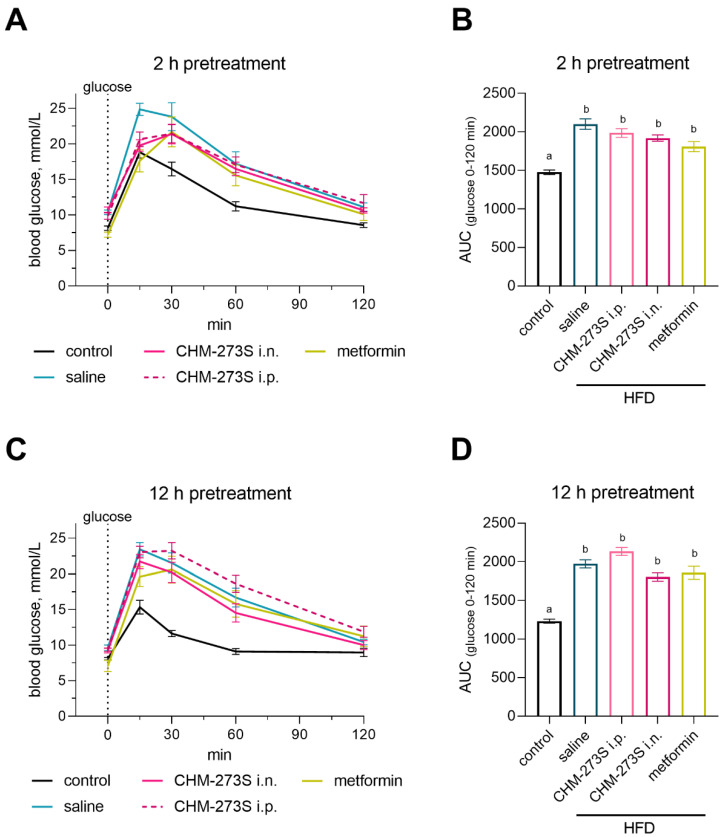
The effects of CHM-273S (5 mg/kg intraperitoneally (i.p.) or intranasally (i.n.), *n* = 12–13 each) and metformin (250 mg/kg perorally (p.o.), *n* = 12–13) pretreatment after 16 weeks of the high-fat diet (HFD) on the oral glucose tolerance test (OGTT) in mice. Blood glucose concentrations were measured by a glucometer after a 12 h fast at 0, 15, 30, 60, and 120 min following gavage with 2 g/kg of an oral glucose (40%) preload (**A**) 2 h or (**C**) 12 h after CHM-273S or metformin treatment. (**B**,**D**) The area under the curve (AUC) for the OGTT in HFD-fed mice after CHM-273S or metformin pretreatment for 2 or 12 h. Compared with the control group, the blood glucose levels were significantly higher in the HFD-fed, saline-treated; HFD-fed, metformin-treated; and HFD-fed, CHM-273S-treated groups when the treatment was administered (**B**) 2 h (F_(4,60)_ = 19.54, *p* < 0.0001) or (**D**) 12 h (F_(4,60)_ = 34.23, *p* < 0.0001) before the OGTT. There was a significant reduction in blood glucose compared with HFD-fed, saline-treated mice in groups pretreated with i.n. CHM-273S or metformin 2 h before the OGTT (F_(4,60)_ = 19.54, *p* < 0.0001). The results are presented as the mean ± standard error of the mean. Different superscript letters indicate significant differences at *p* < 0.05.; one-way analysis of variance with the Holm–Šídák multiple comparisons test.

In the OGTT, we observed increased blood glucose in mice fed the HFD compared with the control animals. A single administration of CHM-273S (5 mg/kg i.n.) 2 but not 12 h before glucose gavage decreased glucose levels similarly to metformin (250 mg/kg p.o.) (Figure 7A–D for the glucose curves for each time point, and Figure 7E for the associated AUC).

After the OGTT, mice were fed the HFD for another 5 weeks (21 weeks in total). From weeks 18 to 21, the animals received CHM-273S (i.n. or i.p.) or metformin (250 mg/kg p.o.). It should be noted that 2 of the 13 metformin-treated mice died during the course of the experiment, after the first and fifth injection of the drug.

**Figure 8:**
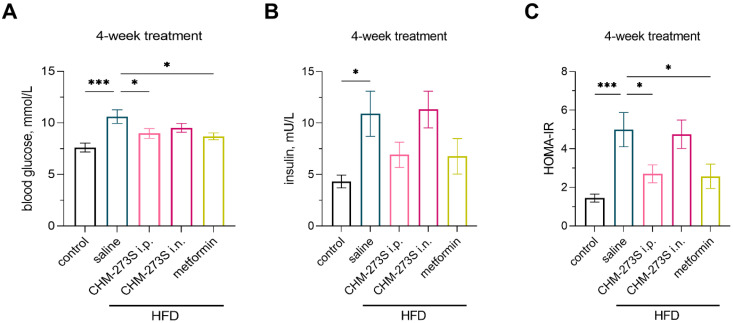
Fasting blood glucose, insulin, and homeostatic model assessment for insulin resistance (HOMA-IR) in high-fat diet (HFD)-fed mice treated for 4 weeks with CHM-273S (5 mg/kg intraperitoneally (i.p.) or intranasally (i.n.), *n* = 10 each) or metformin (250 mg/kg perorally (p.o.), *n* = 10). (**A**) CHM-273S i.p. and metformin significantly reduced blood glucose levels in comparison with HFD-fed, saline-treated animals (F_(4,45)_ = 5.36, *p* = 0.0015). (**B**) Plasma insulin was significantly higher in HFD-fed mice (*n* = 10) compared with control animals (*n* = 10; F_(4,45)_ = 3.58, *p* = 0.013), but neither CHM-273S nor metformin significantly reduced insulin levels compared with the HFD-fed, saline-treated group. (**C**) HOMA-IR was restored to the control values in HFD-fed mice when treated with i.p. CHM-273 or metformin (F_(4,45)_ = 5.98, *p* = 0.0007). The results are presented as the mean ± standard error of the mean. * *p* < 0.05, *** *p* < 0.001, versus the HFD-fed, saline-treated group; one-way analysis of variance with the Holm–Šídák multiple comparisons test.

Analysis of blood glucose and insulin revealed significantly higher levels of both analytes in HFD-fed mice (Figure 8A,B). Hyperglycemia induced by HFD was abolished by chronic i.p. CHM-273S or metformin; there were no treatment-related effects on insulin concentrations (Figure 8A,B). HOMA-IR was also reduced to control values in HFD-fed groups treated with i.p. CHM-273S or metformin (Figure 8C).

**Figure 9:**
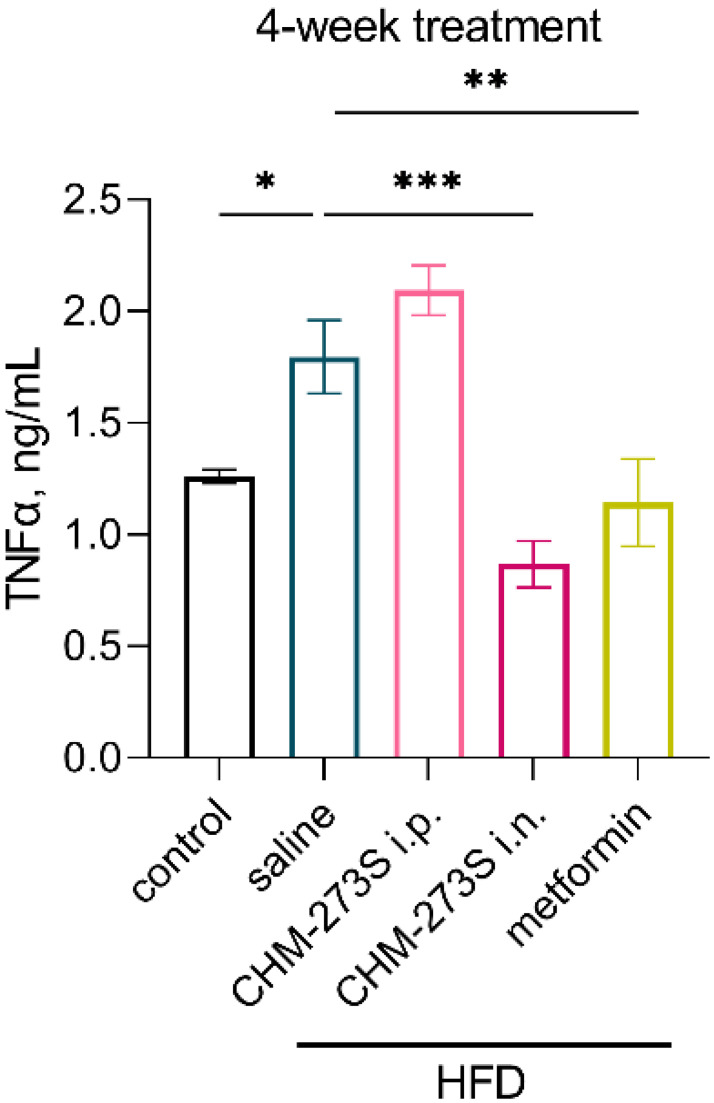
The results of tumor necrosis alpha (TNFα) immunoassay in mouse serum. The increased circulating TNFα level in the serum of high-fat diet (HFD)-fed mice was abolished by 4-week treatment with CHM-273S (5 mg/kg intranasally, *n* = 10) or metformin (250 mg/kg perorally, *n* = 10) (F_(4,45)_ = 12.44, *p* < 0.0001). The results are presented as the mean ± standard error of the mean. * *p* < 0.05, ** *p* < 0.01, *** *p* < 0.001, versus the HFD-fed, saline-treated group; one-way analysis of variance with the Holm–Šídák multiple comparisons test.

Because obesity and T2D are accompanied by systemic inflammation, we analyzed blood concentrations of the proinflammatory cytokine TNFα. Chronic i.n. CHM-273S or metformin treatment potently reduced the HFD-evoked increase in TNFα in mouse serum (Figure 9).

**Figure 10:**
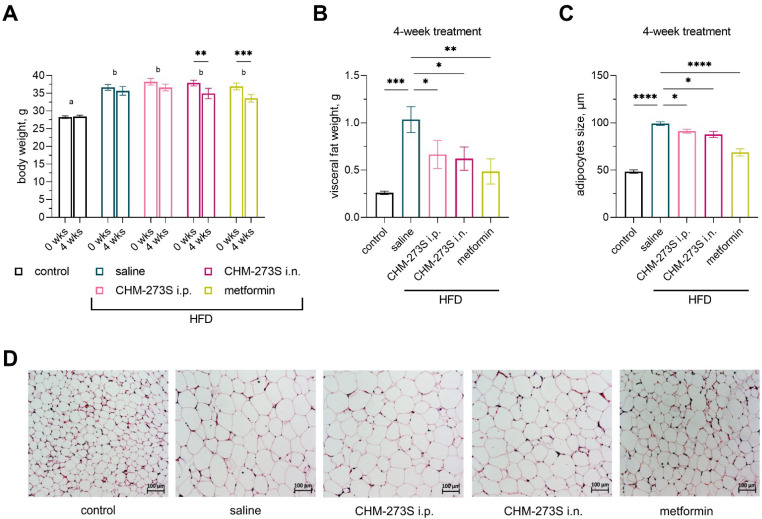
Body weight, visceral fat pad weight, and histological analysis of visceral adipocytes of mice fed the high-fat diet (HFD) after 4 weeks of treatment with CHM-273S (5 mg/kg, intraperitoneally (i.p.) or intranasally (i.n.), *n* = 10 each) or metformin (250 mg/kg perorally (p.o.), *n* = 10). (**A**) Body weight showed significant treatment (F_(4,45)_ = 18.4, *p* < 0.0001) and time (F_(1,45)_ = 23.31, *p* < 0.0001) main effects and a significant treatment × time interaction (F_(4,45)_ = 3.21, *p* = 0.02). HFD-fed mice had excess weight compared with control animals fed a standard diet. The body weight of animals that received i.p. CHM-273S or metformin significantly decreased after 4-week treatment. (**B**) Excess visceral fat surrounding the testes and (**C**) the enlargement of adipocytes in HFD-fed mice was less pronounced when animals were treated with either CHM-273S (i.n. or i.p.) or metformin (F_(4,45)_ = 5.7, *p* = 0.0009 and F_(4,45)_ = 57.7, *p* < 0.0001, respectively). The results are presented as the mean ± standard error of the mean. Different superscript letters indicate significant differences at *p* < 0.05. * *p* < 0.05, ** *p* < 0.01, *** *p* < 0.001, **** *p* < 0.0001 versus the HFD-fed, saline-treated group; repeated measures analysis of variance (ANOVA) (body weight) or one-way ANOVA (visceral fat pad weight and adipocyte size) with the Holm–Šídák multiple comparisons test. (**D**) Representative histological images of visceral adipocytes.

There was significant weight loss for chronic i.n. CHM-273S or metformin treatment over 4 weeks: 7.8% (±10%) and 9% (±7%), respectively (Figure 10A). We found that CHM-273S (i.n. or i.p.) and metformin reduced the adipose tissue mass surrounding the testis and the size of the adipocytes compared with HFD-fed, saline-treated mice (Figure 10B–D).

## 4. Discussion

We have characterized a novel, previously undescribed peptide with potential implications in treating metabolic diseases, such as obesity and T2D. We used HPLC/MS-MS to identify several peptides with previously undescribed functions in *B. taurus* milk hydrolysate and tested their biological activity. We found that CHM-273, as well as its pharmacophores CHM-273S and CHM-273L, enhanced *Irs2* transcription in primary murine fibroblasts. Because CHM-273 and CHM-273L contain CHM-273S, we suggest that CHM-273S provides the peptides’ effect. Upregulation of insulin receptor signaling is a promising approach to treat obesity and diabetes [28,30], and the action of glucose-lowering agents, such as GLP-1 and extendin-4, is usually accompanied by the increased *Irs2* expression [28,30,31].

Considering the potential involvement of the CHM-273S peptide in insulin signaling regulation, typical for appetite-controlling drugs, we decided to test the acute effects of CHM-273S on the appetite of mice in home-cage conditions. CHM-273S was administered intranasally as it is a more comfortable route than injections and is considered to be beneficial in the case of perspective prediabetes [59] and obesity [60] treatment. Additionally, i.n. administration seems to be an efficient way to deliver peptide agents directly to the brain (for instance, it was reported for GLP-1 receptor agonists [61,62] and galanin-like peptide [63]) that are critical for appetite regulation. CHM-273S pretreatment effectively reduced free food intake. Leptin, our comparison drug, failed to produce an acute anorexigenic effect. Previous studies have indicated that chronic leptin reduces appetite in animals [64,65], but a single leptin administration was probably insufficient to regulate the appetite of mice over the 90 min experiment [66].

The hypothalamus may play a key role in the early onset of diabetes because it is involved in the control of glucose homeostasis and energy balance [67,68,69], as well as appetite regulation by leptin [69,70] and insulin [71]. There were more p-STAT3-immunoreactive cells in the hypothalamus after CHM-272S or leptin treatment, suggesting that appetite regulation might be partly attributed to STAT3 upregulation in a manner similar to leptin, which is known to stimulate STAT3 activation in POMC neurons [69,70].

We tested the potential of CHM-273 to regulate carbohydrate metabolism in the HSD-fed rats. I.p. CHM-273S administration was applied as the model of the subcutaneous route that is the most common for peptide-based glucose-lowering drugs of modern generations, such as GLP-1 receptor agonists [72]. Five weeks of the HSD produced glucose intolerance and insulin resistance without changes in body weight. A single administration of CHM-273S significantly reduced blood glucose in the OGTT when the peptide was administered 0, 2, or 12 h before the test. CHM-273S pretreatment also produced a marked reduction in HOMA-IR, indicating the potential ability of the peptide to alleviate insulin resistance state. Although HOMA-IR appears to be an indirect measure of insulin resistance and in some cases does not allow strong conclusions regarding the insulin sensitivity state [73], it demonstrates high correlation with the presence of insulin resistance in multiple diet-induced rodent models and is considered as a robust test in this case [74,75]. Analysis of p-Akt (Ser473), p-Akt (Thr308), and p-p70S6K (Thr421/Ser424) protein expression in the liver revealed a significant reduction in p-Akt (Thr308) in the hepatocytes of HSD rats, suggesting impaired insulin signaling. Studies of metabolic conditions associated with insulin resistance have shown decreased Akt phosphorylation at Thr308 and Ser473 in response to insulin [76,77,78]. Low p-Akt levels are also characteristic of animal models of metabolic dysfunctions, such as HFD [79] and transgenic models [80]. Insulin-stimulated Akt phosphorylation at Thr308 and Ser473 was enhanced by CHM-273S administration. Such an effect might contribute to the peptide’s positive regulation of blood glucose and insulin resistance. Therapeutics with the potential to alter insulin sensitivity normalize or induce p-Akt levels, including metformin [78], glucagon receptor agonists [81], and GLP1 agonists [82]. Neither diet nor the peptide affected p-p70S6K (Thr421/Ser424) protein levels in the liver. This kinase is a downstream effector of the PI3K/Akt/mTOR pathway activated by an ordered cascade of Ser/Thr phosphorylation events (Thr421/Ser424 in particular) and is best known for its regulatory roles in protein synthesis and cell growth. Apart from that, p70S6K is involved in the negative feedback loop, inhibiting IRS/Akt activity, leading to insulin resistance in pathological conditions such as HFD and T2D, where hyperactivation of p70S6K takes place in the liver and muscle [83,84]. Although CHM-273S increased Akt activity, it had no effect on p-p70S6K in hepatocytes, suggesting that it positively regulates insulin signaling and alleviates HSD-induced insulin resistance.

In the current study, we observed a 46% excess weight gain in animals fed the HFD for 16 weeks (45% fat content by weight versus 9% fat in regular chow), which can be considered severe obesity in rodents [41]. We chose the most prescribed drug for T2D, metformin, to compare with CHM-273S. In our experiment, two mice died after first and fifth administration of the drug. Previous studies have reported high-dose metformin toxicity in mice, with a single dose of 600 mg/kg being lethal [85,86]. However, low-dose (150 mg/kg) metformin lacks hypoglycemic effect and fails to activate hepatic AMPK [87]. We chose a dose of 250 mg/kg as effective according to the literature, with or without reported toxicity [88,89]. In the most comprehensive HFD study, we administered CHM-273S both i.n. and i.p. to evaluate the efficiency of both routes and to support our previous findings.

In the OGTT, mice showed glucose intolerance, which was corrected by acute i.n. CHM-273S administered 2 h prior to the glucose gavage. This effect was similar to that observed after p.o. metformin, and in accordance with previous reports [90,91]. After 21 weeks, HFD-fed mice had high fasting glucose, insulin, and HOMA-IR. Based on previous studies, acute and chronic metformin administration has a glucose-lowering effect in C57Bl/6 mice kept on HFD [90,91]. Chronic i.p. CHM-273S, in a similar manner to metformin, potently alleviated glucose and HOMA-IR indices. HFD-induced obesity in mice was accompanied by an increased visceral fat pad weight and adipocyte enlargement. Excessive visceral fat accumulation is strongly associated with obesity-related complications such as T2D and CVD [92,93]. Four-week treatment with CHM-273S resulted in a partial weight loss in mice, loss of visceral fat, and adipocyte shrinkage. The same effects on total weight and visceral fat mass were observed in mice chronically treated with metformin, findings consistent with previous studies [94,95]. The influence of CHM-273S on adipocyte size was similar to that of metformin, although less pronounced.

Cross-talk between metabolic and inflammatory signaling pathways may play an essential role in the development of insulin resistance and the pathophysiology of major public health problems such as diabetes and obesity [27,92]. Recent studies have shown that metformin not only improves chronic inflammation by improving metabolic parameters, but also has a direct anti-inflammatory effect [96,97]. Our results showed that HFD-fed animals had higher TNFα levels in the serum, suggesting the development of low-grade systemic inflammation. Chronic CHM-273S i.n. administration abolished the elevated TNFα levels in a manner similar to metformin.

Our experiments revealed a beneficial effect of acute and chronic administration of the novel peptide CHM-273S in animal models of diet-induced metabolic diseases. CHM-273S potently induced insulin signaling pathways and decreased the inflammatory response. The central action of CHM-273S includes the regulation of appetite. The effects of CHM-273S—induction of IRS and AKT, activation of STAT3 in the hypothalamus, blood glucose reduction, and weight loss—suggest its profile of action typical for modern anti-diabetic agents. This should be thoroughly studied in other in vitro and in vivo models.

Another point of interest is the potential influence of CHM-273S on the melanocortin system through activation of hypothalamic STAT3. Leptin-stimulated STAT3 activation in POMC-expressing neurons promotes energy expenditure and suppresses food intake. However, exogenous leptin therapy lacks efficacy due to obesity-induced leptin resistance. Other mechanisms activating POMC neurons, such as GLP-1 receptor and AMY2 agonists, are of clinical interest. Our next step is to determine whether STAT3 activation and appetite regulation by CHM-273S are carried out through the activation of POMC neurons. Taken together, our results suggest that the CHM-273S peptide should undergo further development as a drug candidate for obesity and T2D treatment. Additionally, because short peptides such as CHM-273S typically have adequate safety and tolerability profiles, they are a potential risk-free treatment option for borderline conditions, such as prediabetes.

## 5. Conclusions

A novel peptide, CHM-273S, regulates appetite, improves glucose tolerance and insulin resistance, and induces weight loss in animal models of diet-induced metabolic diseases. CHM-273S was efficacious even after a single administration. The peptide nature of CHM-273S suggests that it would have adequate safety and tolerability profiles when administered chronically. The CHM-273S mechanisms of action involve canonical IRS signaling with activation of Akt, leptin-like STAT3 signaling in the hypothalamus, and suppression of low-grade inflammation by reducing circulating TNFα. Additional characterization of CHM-273S is necessary to ascertain its direct and indirect targets, as well as its therapeutic potential as an anti-obesity and anti-diabetic drug.

## Figures and Tables

**Table 1:** The origin and amino acid sequences of the tested peptides.

Amino Acid Sequence	Source of Peptide		
SKDIGSESTEDQAME	CasA1		**CHM-273 fragments**
REQLSTSEEN	CasA2		Amino acid sequence
NKKIEKF	CasB		DLSKEP
VQVTSTAV	CasK		SISRE
LIVTQTMKGLD	LacB		SKEPSIS (CHM-273L)
WENGECAQK	LacB		DLSK
DLSKEPSISRE (CHM-273)	GlyCam1		LSKE
NKPEDETHL	GlyCam1	→	SKEP
FEVVKT	GlyCam1		ISRE
NLENTVK	GlyCam1		PSIS
AGGPGAPADPGRPT	PIGR		SISR
SNVQSPD	OSTP		EPSI (CHM-273S)
SHIESEEMHD	OSTP		KEPS
HKSEEDKHL	OSTP		

**Table 2:** Body weight and fasting blood glucose levels in rats fed the high-sucrose diet (HSD). From day 1 of the experiment, rats from the HSD group (*n* = 45) were presented with two drinkers: one with water and one with 30% sucrose in water. Blood glucose concentrations were measured by a glucometer after a 12 h fast. The results are presented as the mean ± standard deviation. * *p* < 0.05 versus the control group on the same day; ^a^ *p* < 0.05 versus day 8, ^b^ *p* < 0.05 versus day 15, ^c^ *p* < 0.05 versus day 22 within a group; Mann–Whitney test, multiple comparisons controlled by two-stage linear step-up procedure of Benjamini, Krieger, and Yekutieli, at a Q threshold of 0.05.

DAY OF EXPERIMENT	BODY WEIGHT (G)				BLOOD GLUCOSE (MMOL/L)			
	Control (*n* = 5)		HSD (*n* = 45)		Control (*n* = 5)		HSD (*n* = 45)	
**0**	402.2	±27.8	415.0	±33.6				
**8**	431.2	±26.0	434.3	±35.6	4.8	±0.2	4.7	±0.4
**15**	436.6	±30.2	451.5	±41.3	5	±0.7	4.7	±0.5
**22**	449	±25.6	452.1	±44.3	5.3	±0.6	5.4 ^ab^	±0.6
**28**	456.4	± 24.0	467.0	± 47.7	5.4	± 0.6	6.1 * ^abc^	± 0.5

**Table 3:** Body weight of mice fed the high-fat diet (HFD) or the standard diet for 16 weeks. The results are presented as the mean ± standard deviation. * *p* < 0.05 versus the control group; Mann–Whitney test, multiple comparisons controlled by two-stage linear step-up procedure of Benjamini, Krieger, and Yekutieli, at a Q threshold of 0.05.

WEEKS	CONTROL (*N* = 25)		HFD (*N* = 100)	
**0**	20.5	±1	20.1	±1.1
**16**	28.9	±1.6	37.6 *	±3.6
**% OF WEIGHT GAIN**	41%	±7.8%	87.5%	±20.1%

## Data Availability

Data are contained within the article.

## Supplementary Materials

The following supporting information can be downloaded at: https://www.mdpi.com/article/10.3390/pharmaceutics14102088/s1, Figure S1: Differential expression of the selected genes in primary murine fibroblasts after incubation with total proprietary milk hydrolysate.

## References

[1] Fitzgerald R.J., A Murray B. (2006). Bioactive peptides and lactic fermentations. Int. J. Dairy Technol..

[2] Schellekens H., Nongonierma A.B., Clarke G., van Oeffelen W.E., FitzGerald R.J., Dinan T.G., Cryan J. (2014). Milk protein-derived peptides induce 5-HT2C-mediated satiety in vivo. Int. Dairy J..

[3] Park Y.W., Nam M.S. (2015). Bioactive Peptides in Milk and Dairy Products: A Review. Korean J. Food Sci. Anim. Resour..

[4] Torres-Fuentes C., Schellekens H., Dinan T.G., Cryan J.F. (2014). A natural solution for obesity: Bioactives for the prevention and treatment of weight gain. A review. Nutr. Neurosci..

[5] Howick K., Wallace-Fitzsimons S.E., Kandil D., Chruścicka B., Calis M., Murphy E., Murray B.A., Fernandez A., Barry K.M., Kelly P.M. (2018). A Dairy-Derived Ghrelinergic Hydrolysate Modulates Food Intake In Vivo. Int. J. Mol. Sci..

[6] Lau J.L., Dunn M.K. (2018). Therapeutic peptides: Historical perspectives, current development trends, and future directions. Bioorganic. Med. Chem..

[7] Sartorius T., Weidner A., Dharsono T., Boulier A., Wilhelm M., Schön C. (2019). Postprandial Effects of a Proprietary Milk Protein Hydrolysate Containing Bioactive Peptides in Prediabetic Subjects. Nutrients.

[8] McGregor R.A., Poppitt S.D. (2013). Milk protein for improved metabolic health: A review of the evidence. Nutr. Metab..

[9] Gregersen S., Bystrup S., Overgaard A., Jeppesen P.B., Thorup A.C.S., Jensen E., Hermansen K. (2013). Effects of Whey Proteins on Glucose Metabolism in Normal Wistar Rats and Zucker Diabetic Fatty (ZDF) Rats. Rev. Diabet. Stud..

[10] Neder Morato P., Lollo P.C.B., Moura C.S., Batista T.M., Camargo R.L., Carneiro E.M., Amaya-Farfan J. (2013). Whey Protein Hydrolysate Increases Translocation of GLUT-4 to the Plasma Membrane Independent of Insulin in Wistar Rats. PLoS ONE.

[11] Gaudel C., Nongonierma A.B., Maher S., Flynn S., Krause M., Murray B.A., Kelly P.M., Baird A.W., Fitzgerald R.J., Newsholme P. (2013). A Whey Protein Hydrolysate Promotes Insulinotropic Activity in a Clonal Pancreatic β-Cell Line and Enhances Glycemic Function in ob/ob Mice. J. Nutr..

[12] Wang K., Fu Z., Li X., Hong H., Zhan X., Guo X., Luo Y., Tan Y. (2022). Whey Protein Hydrolysate Alleviated Atherosclerosis and Hepatic Steatosis by Regulating Lipid Metabolism in ApoE-/- Mice Fed a Western Diet. Food Research International.

[13] Iwasa M., Takezoe S., Kitaura N., Sutani T., Miyazaki H., Aoi W. (2021). A milk casein hydrolysate-derived peptide enhances glucose uptake through the AMP-activated protein kinase signalling pathway in skeletal muscle cells. Exp. Physiol..

[14] D’Souza K., Mercer A., Mawhinney H., Pulinilkunnil T., Udenigwe C.C., Kienesberger P.C. (2020). Whey Peptides Stimulate Differentiation and Lipid Metabolism in Adipocytes and Ameliorate Lipotoxicity-Induced Insulin Resistance in Muscle Cells. Nutrients.

[15] Nongonierma A.B., FitzGerald R.J. (2013). Inhibition of dipeptidyl peptidase IV (DPP-IV) by tryptophan containing dipeptides. Food Funct..

[16] Kondrashina A., Brodkorb A., Giblin L. (2020). Dairy-derived peptides for satiety. J. Funct. Foods.

[17] Ezquerra E.A., Vázquez J.M.C., Barrero A.A. (2008). Obesity, Metabolic Syndrome, and Diabetes: Cardiovascular Implications and Therapy. Rev. Española De Cardiol..

[18] Sanchez-Rangel E., Inzucchi S.E. (2017). Metformin: Clinical use in type 2 diabetes. Diabetologia.

[19] Drugs@FDA: FDA-Approved Drugs. https://www.accessdata.fda.gov/scripts/cder/daf/index.cfm?event=overview.process&ApplNo=215866.

[20] Dahl D., Onishi Y., Norwood P., Huh R., Bray R., Patel H., Rodríguez Á. (2022). Effect of Subcutaneous Tirzepatide vs Placebo Added to Titrated Insulin Glargine on Glycemic Control in Patients With Type 2 Diabetes. JAMA.

[21] Jastreboff A.M., Aronne L.J., Ahmad N.N., Wharton S., Connery L., Alves B., Kiyosue A., Zhang S., Liu B., Bunck M.C. (2022). Tirzepatide Once Weekly for the Treatment of Obesity. New Engl. J. Med..

[22] Yao F., MacKenzie R.G. (2010). Obesity Drug Update: The Lost Decade?. Pharmaceuticals.

[23] Markham A. (2021). Setmelanotide: First Approval. Drugs.

[24] Liu H., Du T., Li C., Yang G. (2021). STAT3 phosphorylation in central leptin resistance. Nutr. Metab..

[25] Péterfi Z., Szilvásy-Szabó A., Farkas E., Ruska Y., Pyke C., Knudsen L.B., Fekete C. (2020). Glucagon-Like Peptide-1 Regulates the Proopiomelanocortin Neurons of the Arcuate Nucleus both Directly and Indirectly via Presynaptic Action. Neuroendocrinology.

[26] Boccia L., Gamakharia S., Coester B., Whiting L., Lutz T.A., Le Foll C. (2020). Amylin brain circuitry. Peptides.

[27] Gao Z., Hwang D., Bataille F., Lefevre M., York D., Quon M.J., Ye J. (2002). Serine Phosphorylation of Insulin Receptor Substrate 1 by Inhibitor κB Kinase Complex. J. Biol. Chem..

[28] White M.F. (2003). Insulin Signaling in Health and Disease. Science.

[29] Huang X., Liu G., Guo J., Su Z. (2018). The PI3K/AKT pathway in obesity and type 2 diabetes. Int. J. Biol. Sci..

[30] Lin X., Taguchi A., Park S., A Kushner J., Li F., Li Y., White M.F. (2004). Dysregulation of insulin receptor substrate 2 in β cells and brain causes obesity and diabetes. J. Clin. Investig..

[31] Zander M., Madsbad S., Madsen J.L., Holst J.J. (2002). Effect of 6-week course of glucagon-like peptide 1 on glycaemic control, insulin sensitivity, and β-cell function in type 2 diabetes: A parallel-group study. Lancet.

[32] Rui L., Yuan M., Frantz D., Shoelson S., White M.F. (2002). SOCS-1 and SOCS-3 Block Insulin Signaling by Ubiquitin-mediated Degradation of IRS1 and IRS2. J. Biol. Chem..

[33] Pinent M., González-Abuín N., Blay M., Ardévol A., Mauricio D. (2016). Chapter 16—Dietary Proanthocyanidin Modulation of Pancreatic β Cells: Molecular Aspects. Molecular Nutrition and Diabetes.

[34] Stanhope K.L. (2015). Sugar consumption, metabolic disease and obesity: The state of the controversy. Crit. Rev. Clin. Lab. Sci..

[35] Wong S.K., Chin K.-Y., Suhaimi F.H., Fairus A., Ima-Nirwana S. (2016). Animal models of metabolic syndrome: A review. Nutr. Metab..

[36] Hill J.O., Melanson E.L., Wyatt H.T. (2000). Dietary Fat Intake and Regulation of Energy Balance: Implications for Obesity. J. Nutr..

[37] Schrauwen P., Westerterp K.R. (2000). The role of high-fat diets and physical activity in the regulation of body weight. Br. J. Nutr..

[38] Jéquier E. (2002). Pathways to obesity. Int. J. Obes. Relat. Metab. Disord..

[39] French S., Robinson T. (2003). Fats and food intake. Curr. Opin. Clin. Nutr. Metab. Care.

[40] Buettner R., Schölmerich J., Bollheimer L.C. (2012). High-fat Diets: Modeling the Metabolic Disorders of Human Obesity in Rodents. Obesity.

[41] Hariri N., Thibault L. (2010). High-fat diet-induced obesity in animal models. Nutr. Res. Rev..

[42] Collins S., Martin T.L., Surwit R.S., Robidoux J. (2004). Genetic vulnerability to diet-induced obesity in the C57BL/6J mouse: Physiological and molecular characteristics. Physiol. Behav..

[43] Inui A. (2003). Obesity—A chronic health problem in cloned mice?. Trends Pharmacol. Sci..

[44] Seluanov A., Vaidya A., Gorbunova V. (2010). Establishing Primary Adult Fibroblast Cultures From Rodents. J. Vis. Exp..

[45] Ge T.T., Yao X.X., Zhao F.L., Zou X.H., Yang W., Cui R.J., Li B.J. (2020). Role of leptin in the regulation of food intake in fasted mice. J. Cell. Mol. Med..

[46] Cady G., Landeryou T., Garratt M., Kopchick J.J., Qi N., Garcia-Galiano D., Elias C.F., Myers M.G., Miller R.A., Sandoval D.A. (2017). Hypothalamic growth hormone receptor (GHR) controls hepatic glucose production in nutrient-sensing leptin receptor (LepRb) expressing neurons. Mol. Metab..

[47] Vasanji Z., Cantor E.J.F., Juric D., Moyen M., Netticadan T. (2006). Alterations in cardiac contractile performance and sarcoplasmic reticulum function in sucrose-fed rats is associated with insulin resistance. Am. J. Physiol. Physiol..

[48] Dutta K., Podolin D.A., Davidson M.B., Davidoff A.J. (2001). Cardiomyocyte Dysfunction in Sucrose-Fed Rats Is Associated With Insulin Resistance. Diabetes.

[49] Wold L.E., Dutta K., Mason M.M., Ren J., Cala S.E., Schwanke M.L., Davidoff A.J. (2005). Impaired SERCA function contributes to cardiomyocyte dysfunction in insulin resistant rats. J. Mol. Cell. Cardiol..

[50] Gerbaix M., Metz L., Ringot E., Courteix D. (2010). Visceral fat mass determination in rodent: Validation of dual-energy x-ray absorptiometry and anthropometric techniques in fat and lean rats. Lipids Health Dis..

[51] Mistry A.M., Swick A.G., Romsos D.R. (1997). Leptin rapidly lowers food intake and elevates metabolic rates in lean and ob/ob mice. J. Nutr..

[52] Sadagurski M., Nofech-Mozes S., Weingarten G., White M.F., Kadowaki T., Wertheimer E. (2007). Insulin receptor substrate 1 (IRS-1) plays a unique role in normal epidermal physiology. J. Cell. Physiol..

[53] Sadagurski M., Weingarten G., Rhodes C.J., White M.F., Wertheimer E. (2005). Insulin Receptor Substrate 2 Plays Diverse Cell-specific Roles in the Regulation of Glucose Transport. J. Biol. Chem..

[54] Monaco S., Illario M., Rusciano M.R., Gragnaniello G., Di Spigna G., Leggiero E., Pastore L., Fenzi G., Rossi G., Vitale M. (2009). Insulin stimulates fibroblast proliferation through calcium-calmodulin-dependent kinase II. Cell Cycle.

[55] Malyshev A.V., Sukhanova I.A., Zlobin A.S., Gedzun V.R., Pavshintsev V.V., Vasileva E.V., Zalevsky A.O., Doronin I.I., Mitkin N.A., Golovin A.V. (2021). In silico Screening and Behavioral Validation of a Novel Peptide, LCGA-17, With Anxiolytic-Like Properties. Front. Neurosci..

[56] Malyshev A.V., Sukhanova I.A., Ushakova V.M., Zorkina Y.A., Abramova O.V., Morozova A.Y., Zubkov E.A., Mitkin N.A., Pavshintsev V.V., Doronin I.I. (2022). Peptide LCGA-17 Attenuates Behavioral and Neurochemical Deficits in Rodent Models of PTSD and Depression. Pharmaceuticals.

[57] West J.A., Tsakmaki A., Ghosh S.S., Parkes D.G., Grønlund R.V., Pedersen P.J., Maggs D., Rajagopalan H., Bewick G.A. (2021). Chronic peptide-based GIP receptor inhibition exhibits modest glucose metabolic changes in mice when administered either alone or combined with GLP-1 agonism. PLoS ONE.

[58] Hampe L., Xu C., Harris P.W.R., Chen J., Liu M., Middleditch M., Radjainia M., Wang Y., Mitra A.K. (2017). Synthetic peptides designed to modulate adiponectin assembly improve obesity-related metabolic disorders: De-signed Peptides Counter Obesity-Related Disorders. J. Cereb. Blood Flow Metab..

[59] Dholakia J., Prabhakar B., Shende P. (2021). Strategies for the delivery of antidiabetic drugs via intranasal route. Int. J. Pharm..

[60] Keller L.-A., Merkel O., Popp A. (2021). Intranasal drug delivery: Opportunities and toxicologic challenges during drug development. Drug Deliv. Transl. Res..

[61] Banks W.A., During M.J., Niehoff M.L. (2004). Brain Uptake of the Glucagon-Like Peptide-1 Antagonist Exendin(9-39) after Intranasal Administration. J. Pharmacol. Exp. Ther..

[62] Rykalina N.V., Askerova E.V., Bulushova N.V., Kozlov D.G. (2020). Intranasal Human Recombinant Modified Glucagon-Like Peptide-1: High Antihyperglycemic Activity and Duration of Action in Mice. Bull. Exp. Biol. Med..

[63] Kageyama H., Shiba K., Hirako S., Wada N., Yamanaka S., Nogi Y., Takenoya F., Nonaka N., Hirano T., Inoue S. (2016). Anti-obesity effect of intranasal administration of galanin-like peptide (GALP) in obese mice. Sci. Rep..

[64] Sharma A., Bartell S.M., Baile C.A., Chen B., Podolsky R.H., McIndoe R.A., She J.-X. (2010). Hepatic Gene Expression Profiling Reveals Key Pathways Involved in Leptin-Mediated Weight Loss in ob/ob Mice. PLoS ONE.

[65] Li X., Wu X., Camacho R., Schwartz G.J., Leroith D. (2011). Intracerebroventricular Leptin Infusion Improves Glucose Homeostasis in Lean Type 2 Diabetic MKR Mice via Hepatic Vagal and Non-Vagal Mechanisms. PLoS ONE.

[66] Fan X., Bradbury M.W., Berk P.D. (2003). Leptin and Insulin Modulate Nutrient Partitioning and Weight Loss in ob/ob Mice through Regulation of Long-Chain Fatty Acid Uptake by Adipocytes. J. Nutr..

[67] Banas S.M., Rouch C., Kassis N., Markaki E.M., Gerozissis K. (2008). A Dietary Fat Excess Alters Metabolic and Neuroendocrine Responses Before the Onset of Metabolic Diseases. Cell. Mol. Neurobiol..

[68] Thorens B. (2011). Brain glucose sensing and neural regulation of insulin and glucagon secretion. Diabetes Obes. Metab..

[69] Burgos-Ramos E., González-Rodríguez Á., Canelles S., Baquedano E., Frago L.M., Revuelta-Cervantes J., Gómez-Ambrosi J., Frühbeck G., Chowen J.A., Argente J. (2012). Differential Insulin Receptor Substrate-1 (IRS1)-Related Modulation of Neuropeptide Y and Proopiomelanocortin Expression in Nondiabetic and Diabetic IRS2−/− Mice. Endocrinology.

[70] Kitamura T., Feng Y., Kitamura Y.I., Chua S.C., Xu A.W., Barsh G.S., Rossetti L., Accili D. (2006). Forkhead protein FoxO1 mediates Agrp-dependent effects of leptin on food intake. Nat. Med..

[71] Belgardt B.F., Husch A., Rother E., Ernst M.B., Wunderlich F.T., Hampel B., Klöckener T., Alessi D., Kloppenburg P., Brüning J.C. (2008). PDK1 Deficiency in POMC-Expressing Cells Reveals FOXO1-Dependent and -Independent Pathways in Control of Energy Homeostasis and Stress Response. Cell Metab..

[72] Trujillo J.M., Nuffer W., Smith B.A. (2021). GLP-1 receptor agonists: An updated review of head-to-head clinical studies. Ther. Adv. Endocrinol. Metab..

[73] Ader M., Stefanovski D., Richey J.M., Kim S.P., Kolka C.M., Ionut V., Kabir M., Bergman R.N. (2014). Failure of Homeostatic Model Assessment of Insulin Resistance to Detect Marked Diet-Induced Insulin Resistance in Dogs. Diabetes.

[74] Wallace T.M., Levy J.C., Matthews D.R. (2004). Use and Abuse of HOMA Modeling. Diabetes Care.

[75] Fraulob J.C., Ogg-Diamantino R., Fernandes-Santos C., Aguila M.B., Mandarim-De-Lacerda C.A. (2010). A Mouse Model of Metabolic Syndrome: Insulin Resistance, Fatty Liver and Non-Alcoholic Fatty Pancreas Disease (NAFPD) in C57BL/6 Mice Fed a High Fat Diet. J. Clin. Biochem. Nutr..

[76] Sun W., Bi Y., Liang H., Cai M., Chen X., Zhu Y., Li M., Xu F., Yu Q., He X. (2010). Inhibition of obesity-induced hepatic ER stress by early insulin therapy in obese diabetic rats. Endocrine.

[77] Karlsson H.K., Zierath J.R., Kane S., Krook A., Lienhard G.E., Wallberg-Henriksson H. (2005). Insulin-Stimulated Phosphorylation of the Akt Substrate AS160 Is Impaired in Skeletal Muscle of Type 2 Diabetic Subjects. Diabetes.

[78] Xu H., Zhou Y., Liu Y.X., Ping J., Shou Q.Y., Chen F.M., Ruo R. (2016). Metformin improves hepatic IRS2/PI3K/Akt signaling in insulin-resistant rats of NASH and cirrhosis. J. Endocrinol..

[79] Frøsig C., Jensen T., Jeppesen J., Pehmøller C., Treebak J.T., Maarbjerg S.J., Kristensen J., Sylow L., Alsted T.J., Schjerling P. (2013). AMPK and Insulin Action—Responses to Ageing and High Fat Diet. PLoS ONE.

[80] Lee J.M., Kim Y., Hernández M.A.S., Han Y., Liu R., Park S.W. (2019). BRD7 deficiency leads to the development of obesity and hyperglycemia. Sci. Rep..

[81] Kim T., Holleman C.L., Nason S., Arble D.M., Ottaway N., Chabenne J., Loyd C., Kim J.-A., Sandoval D., Drucker D.J. (2018). Hepatic Glucagon Receptor Signaling Enhances Insulin-Stimulated Glucose Disposal in Rodents. Diabetes.

[82] Tsuboi K., Mizukami H., Inaba W., Baba M., Yagihashi S. (2016). The dipeptidyl peptidase IV inhibitor vildagliptin suppresses development of neuropathy in diabetic rodents: Effects on peripheral sensory nerve function, structure and molecular changes. J. Neurochem..

[83] Tremblay F., Gagnon A., Veilleux A., Sorisky A., Marette A. (2005). Activation of the Mammalian Target of Rapamycin Pathway Acutely Inhibits Insulin Signaling to Akt and Glucose Transport in 3T3-L1 and Human Adipocytes. Endocrinology.

[84] Korsheninnikova E., Van Der Zon G.C.M., Voshol P.J., Janssen G.M., Havekes L.M., Grefhorst A., Kuipers F., Reijngoud D.-J., A Romijn J., Ouwens M. (2006). Sustained activation of the mammalian target of rapamycin nutrient sensing pathway is associated with hepatic insulin resistance, but not with steatosis, in mice. Diabetologia.

[85] Hasan N., Sugimoto K., Yamada K., Morishige J.-I., Ushijima K., Fujimura A., Nagata N., Ando H. (2022). Chronic Treatment with Metformin Has No Disrupting Effect on the Hepatic Circadian Clock in Mice. Medicina.

[86] Martin-Montalvo A., Mercken E.M., Mitchell S.J., Palacios H.H., Mote P.L., Scheibye-Knudsen M., Gomes A.P., Ward T.M., Minor R.K., Blouin M.-J. (2013). Metformin improves healthspan and lifespan in mice. Nat. Commun..

[87] Hassan N.M., Alhossary A.A., Mu Y., Kwoh C.-K. (2017). Protein-Ligand Blind Docking Using QuickVina-W With Inter-Process Spatio-Temporal Integration. Sci. Rep..

[88] Henriksson E., Huber A.-L., Soto E.K., Kriebs A., Vaughan M.E., Duglan D., Chan A.B., Papp S.J., Nguyen M., Afetian M.E. (2017). The Liver Circadian Clock Modulates Biochemical and Physiological Responses to Metformin. J. Biol. Rhythm..

[89] Caton P.W., Kieswich J., Yaqoob M.M., Holness M.J., Sugden M.C. (2011). Metformin opposes impaired AMPK and SIRT1 function and deleterious changes in core clock protein expression in white adipose tissue of genetically-obese db/db mice. Diabetes, Obes. Metab..

[90] Zhou Z.-Y., Ren L.-W., Zhan P., Yang H.-Y., Chai D.-D., Yu Z.-W. (2016). Metformin exerts glucose-lowering action in high-fat fed mice via attenuating endotoxemia and enhancing insulin signaling. Acta Pharmacol. Sin..

[91] Horakova O., Kroupova P., Bardova K., Buresova J., Janovska P., Kopecky J., Rossmeisl M. (2019). Metformin acutely lowers blood glucose levels by inhibition of intestinal glucose transport. Sci. Rep..

[92] Hamdy O., Porramatikul S., Al-Ozairi E. (2006). Bentham Science Publisher Bentham Science Publisher Metabolic Obesity: The Paradox Between Visceral and Subcutaneous Fat. Curr. Diabetes Rev..

[93] Yang Y., Fu M., Li M.-D., Zhang K., Zhang B., Wang S., Liu Y., Ni W., Ong Q., Mi J. (2020). O-GlcNAc transferase inhibits visceral fat lipolysis and promotes diet-induced obesity. Nat. Commun..

[94] Day E.A., Ford R.J., Smith B.K., Mohammadi-Shemirani P., Morrow M.R., Gutgesell R.M., Lu R., Raphenya A.R., Kabiri M., McArthur A.G. (2019). Metformin-induced increases in GDF15 are important for suppressing appetite and promoting weight loss. Nat. Metab..

[95] Coll A.P., Chen M., Taskar P., Rimmington D., Patel S., Tadross J.A., Cimino I., Yang M., Welsh P., Virtue S. (2019). GDF15 mediates the effects of metformin on body weight and energy balance. Nature.

[96] Jing Y., Wu F., Li D., Yang L., Li Q., Li R. (2018). Metformin improves obesity-associated inflammation by altering macrophages polarization. Mol. Cell. Endocrinol..

[97] Bharath L.P., Nikolajczyk B.S. (2021). The intersection of metformin and inflammation. Am. J. Physiol. Physiol..

