# Supplementary material for: The Novel Peptide Chm-273s Has Therapeutic Potential for Metabolic Disorders: Evidence from In Vitro Studies and High-Sucrose Diet and High-Fat Diet Rodent Models
Source: Pharmaceutics. 2022 Sep 30;14(10):2088. doi: 10.3390/pharmaceutics14102088 (PMC9611607; doi:10.3390/pharmaceutics14102088)
Supplement: Supplementary file 1 [file pharmaceutics-14-02088-s001.zip › pharmaceutics-1871354-supplementary.pdf]

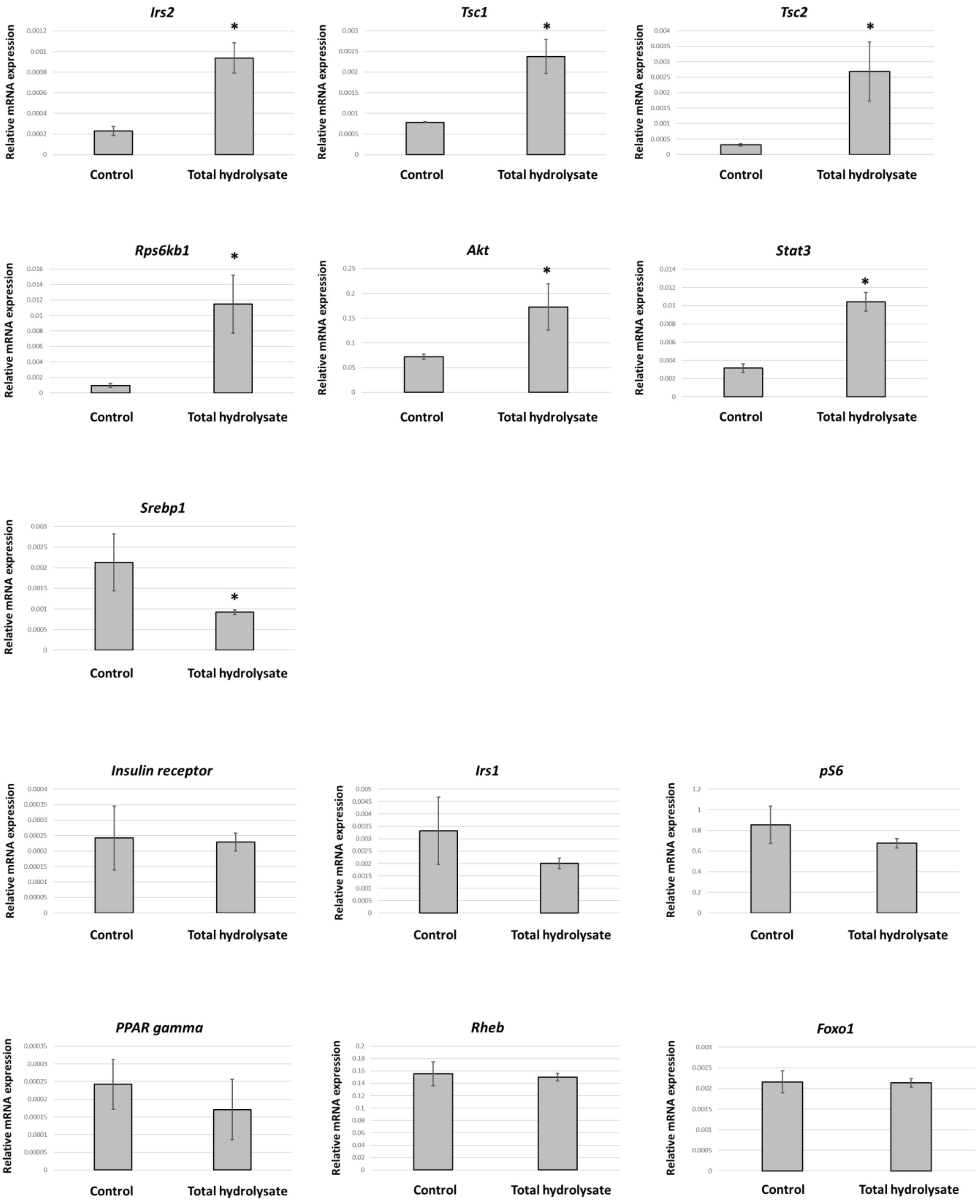

Figure S1. Differential expression of the selected genes in primary murine fibroblasts after incubation with total proprietary milk hydrolysate. For gene expression analysis primary murine fibroblasts were incubated with total milk hydrolysate (0.5 mg/ml) for 24 hours. RNA isolation and reverse transcription–quantitative polymerase chain reaction (RT-qPCR) were performed according to the protocol described in the Materials and Methods section in the main text. Expression levels of *Tsc1*, *Tsc2*, *Rps6kb1*, *Akt*, *Irs2*, *Stat3* and *Srebp1* were significantly triggered by the hydrolysate, these genes were chosen for subsequent screening studies of individual peptides. Expression levels of *insulin receptor*, *Irs1*,

pS6, PPAR gamma, Rheb and *Foxo1* genes were not affected by the hydrolysate. The results are presented as the mean ± SEM of three biological replicates. * $p < 0.05$ versus the control group; nested one-way analysis of variance, discovery by two-stage linear step-up procedure of Benjamini, Krieger, and Yekutieli, at a Q threshold of 0.05.
